# Neurodegenerative Changes to Subiculum Cell Type Morphology in 5xFAD Mice

**DOI:** 10.1002/alz.089846

**Published:** 2025-01-03

**Authors:** Zion MK Smith, Marlene Becerra, Nancy C Zepeda, Luc Pham, Darrin J Lee, Michael S Bienkowski

**Affiliations:** ^1^ Stevens Neuroimaging and Informatics Institute, Keck School of Medicine, University of Southern California, Los Angeles, CA USA; ^2^ USC Center for Integrative Connectomics, Los Angeles, CA USA; ^3^ Laboratory of Neuro Imaging Keck School of Medicine of USC University of Southern California, Los Angeles, CA USA; ^4^ Keck School of Medicine, University of Southern California, Los Angeles, CA USA; ^5^ Zilkha Neurogenetic Institute, Keck School of Medicine, University of Southern California, Los Angeles, CA USA; ^6^ Laboratory of Neuro Imaging, Keck School of Medicine, University of Southern California, Los Angeles, CA USA

## Abstract

**Background:**

Alzheimer’s disease (AD) is a neurological disorder marked by progressive cognitive decline, memory deficits, and neuronal cell loss (Knopman, 2021). A brain region significantly impacted by the progression of AD is the subiculum, a structure responsible for spatial navigation, cognitive processes, and the modulation of emotional and affective behaviors within the hippocampus (Fanselow and Dong, 2010). Although subiculum cell loss has been well‐established as an early indicator of AD (Carlesimo et al., 2015), neurodegenerative changes to cell morphology can only be observed post‐mortem and are limited by tissue histology and staining approaches that cannot completely label neuronal morphology. Using AD mouse models, G‐deleted rabies viral tracers can fluorescently label full projection neuron cell type morphology for 3D reconstruction analysis and identify neurodegenerative changes to soma and dendrites across varying disease timepoints. Understanding morphological changes during disease progression can help identify neuronal cell type susceptibility, which precedes cell death.

**Methods:**

Using 5xFAD mice at 2 and 8 mo. timepoints, we injected G‐deleted rabies viral tracers into the medial mammillary (MM) and lateral hypothalamus (LHA) to retrogradely label MM‐ and LHA‐projecting subiculum neurons. After a 1‐week survival time, brains were extracted, sectioned into 500µm thick coronal sections, processed for SHIELD tissue clearing histology, and 3D imaged using a confocal microscope with 250nm isotropic resolution.

**Results:**

Our findings revealed that G‐deleted rabies vectors fluorescently labeled both ‘healthy’ and ‘sick’ pyramidal neurons within the subiculum, suggesting individual neurons at different stages of neurodegeneration. ‘Sick’ subiculum neurons displayed a variety of distinct alterations in their morphology, including shrunken cell bodies caused by decreased cytoplasm around the nucleus, the emergence of swellings and thinning of dendrites, and complete dendrite collapse. Our analysis of subiculum neurons suggests a progressive timeline of morphological degeneration that precedes cell death within the subiculum.

**Conclusion:**

Our findings have the potential to not only deepen our understanding of the degenerative morphological process in AD but also determine the cellular processes that precede neuronal death, which may in turn lead to developing targeted interventions that could prevent this fate.

